# Relational “Dance” Between Mother and Moderately Preterm Infant at 6 and 9 Months of Correct Age: Possible Risk and Protective Factors

**DOI:** 10.3390/healthcare12222231

**Published:** 2024-11-08

**Authors:** Concetta Polizzi, Serena Iacono Isidoro, Maria Maddalena Di Pasqua, Valentina Fontana, Susanna Marotta, Giovanna Perricone, Margherita Spagnuolo Lobb

**Affiliations:** 1 Department of Psychology, Educational Science and Human Movement (SPPEFF), University of Palermo, 90128 Palermo, Italy; concetta.polizzi@unipa.it (C.P.); 2 Italian Society of Pediatric Psychology (S.I.P.Ped.), 90121 Palermo, Italy; marilenadipasqua77@gmail.com (M.M.D.P.); valentina.fontana@unipa.it (V.F.); susannamarottagestalt@gmail.com (S.M.); 3 Institute for Biomedical Research and Innovation (IRIB), National Research Council of Italy (CNR), 98164 Messina, Italy; 4 Istituto di Gestalt HCC Human Communication Centre Italy, 96100 Siracusa, Italy; margherita.spagnuolo@gestalt.it (M.S.L.); 5 Ombudsman for Children and Adolescents, Municipality of Palermo, 90144 Palermo, Italy; garanteinfanziadolescenza@comune.palermo.it (G.P.)

**Keywords:** “dance steps” model, mother–infant interaction, preterm birth, Gestalt psychotherapy, relational synchrony, observational tool, risk and protective factors

## Abstract

Background/Objectives: This study explores the characteristics of the early mother–infant relationship in a sample of 30 mother–preterm infant dyads between 6 and 9 months, using a phenomenological observational tool called “Dance Steps”. This tool examines the configuration and reciprocity of mother–infant interactions. The study also investigates how sociodemographic factors and maternal functioning variables, such as postnatal depression and perceived social support, may serve as risk or protective factors in the development of these interaction “Steps”. Methods: Observations were conducted through video recordings of face-to-face caregiver–infant interactions during unstructured play sessions at neonatal follow-up visits. The data focused on identifying specific characteristics of reciprocity in the “dance steps”. Results: The results reveal certain features of reciprocity are stable over time, demonstrating synchronicity and attunement in many of the “dance steps”. Other “steps” evolve in response to the child’s developing competence. Sociodemographic factors, particularly the mother’s educational level and the infant’s sex, significantly influence the “Dance”. Postnatal depression negatively affected reciprocity in several steps, especially impacting the infant, whereas perceived social support had a lesser effect. Conclusions: The findings emphasize the importance of supporting mothers of preterm infants to adjust their interactions in ways that promote the child’s developmental competence. This support is essential for fostering physical and emotional closeness during critical developmental transitions.

## 1. Introduction

Preterm birth, defined as childbirth before 37 weeks of pregnancy, represents an important risk condition for the child’s development, as it could direct numerous impairments, including long-term effects, at the expense of different domains of development: from cognitive to socio-emotional, motor, and language [1,2,3,4].

Many of these impairments, especially those affecting emotional regulation and interaction with the environment, are visible from the earliest months of life and are mostly related to the neurophysiological immaturity caused by preterm birth, especially if severe; however, such developmental fragilities are also influenced by socio-environmental factors, first and foremost the early relationship with caregivers and especially the quality of interactions and communication patterns with the mother and father [5,6]. Numerous studies have shown specific difficulties for mothers and fathers in managing interactions with children who are born preterm, compared with parents of children born full term [7,8,9,10,11]. On the other hand, the neurophysiological immaturity of preterm infants often results in them being more passive, less attentive, and less alert during early interactions with their mothers compared to full-term infants [9]. Certainly, these characteristics of the infant’s functioning may lead to the caregiver, particularly the mother, having difficulties in understanding his signals and in finding adequate closeness and distance in the interaction, as well as an adequate supply of stimulation. Numerous studies have observed that mothers of preterm infants tend to engage in highly active and stimulating interactions, contrasting with the interactions of mothers with full-term infants [12].

On the other hand, premature infants often give fewer signals in their interactions with their mothers, such as eye contact and smiles. This can lead mothers to engage in interactions that may feel intrusive, as they might struggle to interpret the meanings behind their infant’s immature behaviors [13].

Furthermore, preterm birth also constitutes a risk condition for parents, particularly in managing their relationship with the child, especially in relation to the essential process of co-regulation [4,14,15]; mother–infant dyads in preterm birth conditions often appear to be characterized by less synchrony [12] and symmetrical co-regulation than dyads in full-term birth [4]. To this extent, greater intrusiveness has often been noted, with little support for the infant’s autonomy [5,16]. In addition, several studies point to the presence of lower sensitivity and responsiveness in mothers of infants who are born preterm [5,11,16], although other studies have not found statistically significant differences with mothers of full-term infants [10].

Additionally, the literature has pointed out that early interactions, especially mother–child interactions, are predictive of children’s behaviors and abilities even in the long term; for example, sensitive behaviors in mothers predict cooperative behaviors in children, and conversely, controlling and unresponsive behaviors in mothers seem to predict passivity or difficulties in the child when relating to the outside world [17].

In this study, the focus and exploration of early mother–preterm infant interactions in the first year of life are based on a specific interpretative model of these interactions, which is of an aesthetic and phenomenological nature, called the “Dance Steps” model [18], as described below.

### 1.1. The “Dance Steps” Model

The “dance steps” model builds upon several theoretical foundations, beginning with attachment theories by Harlow [19], Bowlby [20], and Ainsworth [21], which highlight the caregiver’s role in shaping relational patterns rather than discrete behaviors. Stern’s work [22] shows that infants actively seek interaction, necessitating continuous organization for self-regulation. Neurobiological research, notably the work of Siegel [23], suggests that early relational experiences shape brain structures through implicit memory. In Gestalt therapy, “intentionality of contact” is viewed as key to self-development, with reciprocal caregiver–infant movements fostering a secure sense of self [24].

This model emphasizes reciprocity in interactions, which is essential in clinical practice, where disorders in children often reflect contact-making difficulties. Pediatric psychology underscores that early relational dynamics co-construct an experiential self, involving a balance of predisposition, experience, and potential epigenetic effects [18,25,26]. This study promotes secure attachment and co-regulation through an observational grid that focuses on the aesthetics and phenomenology of reciprocity in caregiver–infant interactions. This aligns with the mutual regulation model [27], in which the caregiver and infant dynamically attune to each other’s cues, fostering attachment and developmental well-being.

The “dance steps” model conceptualizes contact-making as a dynamic, aesthetic process characterized by spontaneity, sensitivity, vitality, grace, and brilliance [28]. These qualities enable natural, responsive, and profound interactions, forming a “dance” of mutual perceptions and intentional movements aimed at achieving a fuller sense of self [29]. Significant bonds are represented as a mutual “going toward” the other, supported by each individual’s vitality [30,31].

Gestalt principles guide the observer to focus on the caregiver–child interaction in the present moment, using aesthetic tools to interpret relational dynamics. By engaging with this “dance”, the observer perceives the deep connection between caregiver and child, rooted in their spontaneous interactions, creating a safe and grounded relational space. These criteria enable the observation of the caregiver–infant dyad’s contact-making experiences and their levels of spontaneity and anxiety. The “dance steps” outline eight sequential steps describing the progression of contact-making interactions between caregivers and infants:

- Building together the sense of the ground: This initial step does not involve expressive movements; rather, it is about intuitively understanding each other’s presence and the context.
- Perceiving one another: This step involves activating relational energy through sensory perceptions.
- Acknowledging one another: Here, both parties recognize and validate the intention behind their interactions, giving meaning to their shared experiences.
- Adjusting to one another: This step requires both parties to attune to each other’s feelings and respond with their own presence and unique approach.
- Taking bold steps together: This involves joint activities that break through existing patterns and lead to new directions, resolving impasses.
- Having fun: The caregiver and infant/child share enjoyable moments, relishing each other’s company and experiencing light-heartedness.
- Connecting: This interaction fosters a sense of mutual accessibility and agency for both parties, enhancing their bond.
- Entrusting oneself to the other/Taking care of the other: The infant/child can trust the caregiver, and the caregiver feels capable of spontaneously caring for the situation. This mutual trust creates a deep sense of intimacy and security.

The “dance steps” model details a progression in caregiver–infant relational dynamics, from establishing a shared foundation to mutual trust, with each step reflecting distinct aspects of their interaction. These steps activate all areas of a child’s development—relational, cognitive, motor, and emotional—enhancing interconnections across these domains [32]. The model highlights reciprocity and mutual adjustment in caregiver–child contact, offering a framework for understanding and assessing these interactions, with applications in clinical practice for fostering healthy relational development.

In pediatric psychology, this interaction provides children with cognitive, emotional, and relational nourishment, identity protection, strategic withdrawal, spiritual exploration, and the ability to transcend immediate experiences, facilitating attunement and resilience against adversity [33,34]. Early on, an active caregiver presence is beneficial; however, as the child develops, it becomes advantageous for the caregiver to take a background role, allowing the child to lead interactions and incorporate new elements. Certain “dance steps”, such as mutual trust and caregiving, benefit from simultaneous and reciprocal engagement, such as when both the mother and child relax together.

### 1.2. Possible Risk and Protective Factors

Early mother–infant interactions are certainly influenced by a range of variables regarding sociodemographic characteristics concerning the mother (such as age, educational level, employment status, and parity), the child’s sex, and the type of delivery, as well as variables related to the mother’s psychological functioning, such as potential postnatal depression and perceived social support.

These variables can, in fact, be defined as risk or protective factors for the quality of early mother–infant interactions.

With specific reference to preterm birth conditions, sociodemographic factors have often been found to have a bearing on early interactions between the preterm-born child and caregiver [35,36]. In this direction, it has been highlighted how higher educational levels in mothers are often related to greater sensitivity during interactions with preterm infants when compared to other mothers [37]; conversely, positive associations have been found between low educational levels, poor employment conditions, and high psychological distress in mothers [17,38,39], which clearly negatively impact the interactions with the child [36,40]. However, research results are not always convergent regarding the relationship between mothers’ educational level and the quality of the bond with the child; for example, some studies have found a negative correlation between the mother’s cultural level and pre- and postnatal bonding [32,41], while other studies have found only a weak association between the two variables [42,43,44]. Therefore, it is important to further explore the impact of maternal education level on the quality of the relationship with the child.

Several studies have examined variables related to maternal obstetrical characteristics (such as parity and mode of delivery) [35,45,46], highlighting the influence of these factors on the early mother–infant relationship. The findings of these studies are not consistent, and our study aims to explore the impact of such variables on the reciprocal behaviors of the mother–infant dyad for children who are born moderately preterm from a longitudinal perspective (at both 6 and 9 months of the child’s corrected age).

An additional variable that can affect the mother–infant interaction is the child’s gender, as research has often associated higher levels of sensitivity and lower parental stress in parents of female infants [35,36]. Similarly, higher scores in attachment have been reported in relation to female infants [47]. However, even with regard to this child-related variable, studies do not always present convergent results, likely due to profound cultural differences [48,49].

Numerous studies on early mother–infant relationships have explored the impact of postpartum depression as well as the influence of perceived social support [50].

Attention to depressive symptoms is relevant due to the negative effects they can have on the child’s development, even in the long term, as they can alter the interactions and the quality of the mother–infant attachment relationship [51,52,53,54,55]. Depressive symptoms are positively associated with less positive emotions and a more dominant attitude of the child as perceived by mothers [56]. Moreover, several studies have shown that women with depressive symptoms experience more difficulties in the relationship with their child during the first year of life compared to women without depression, showing less warmth, less emotional availability, and less closeness to the child [55,56,57,58]. Postpartum depression negatively impacts entire families, including siblings [59].

Preterm birth is often associated with a higher risk of depression and anxiety in both parents [11,37,60,61], particularly in mothers [59,62].

As for perceived social support, both during pregnancy and in the postpartum period, it has been highlighted that this can represent an important protective factor (or, conversely, a risk factor) for the quality of the mother–infant interaction. For example, perceived partner support is often correlated with high maternal sensitivity in interactions with the infant as early as the first 4 months of life [63]. In this sense, the perception of social support, particularly from the partner, has often been indicated in studies in this field as an important positive predictor of the quality of mother–infant interactions starting from the prenatal period, as it reduces stress and promotes greater regulatory capacity in the mother [64,65,66]. Considering the potentially significant effect of perceived social support on the quality of early mother–infant interactions, this study aimed to take into account how supported the involved mothers felt during the first trimester of the postpartum period by family members (particularly the partner), friends, and other significant figures (such as experts, mental health professionals, etc.). Moreover, it explored whether this support influenced the specific reciprocity of the relational dance.

### 1.3. Aims

Starting from these considerations and the “dance steps” model assumed in this study, a longitudinal study was developed with the aim of investigating the characteristics of the early mother–infant preterm-birth relationship through a specific observational tool of a phenomenological nature according to the “dance steps” model.

Specifically, the first aim of this study was to investigate how the moderately preterm mother–child relationship evolves at 6 and 9 months of corrected age. The quality of the mother–child relational “dance” in moderately preterm dyads follows a developmental trajectory linked to the child’s abilities as they transition from 6 to 9 months of their corrected age. As suggested by a previous pilot study [18], the frequency of certain “dance steps” tends to change incrementally or in decrements in relation to the child’s age, reflecting their evolving cognitive, emotional, and relational skills. It is hypothesized that a decrease in some maternal behaviors corresponds to an increase in the child’s behaviors related to the same “dance step”, and vice versa.

The second aim of this study is to explore the potential influence of sociodemographic characteristics of the dyads on the pattern of the relational “dance”. Based on the literature, it is hypothesized that variables such as maternal age, parity, mode of delivery, child’s sex, maternal educational level, and employment status may impact the quality of the relational patterns established with the child.

Finally, this study aims to assess possible correlations between the activation of “dance steps” at 6 and 9 months and maternal postnatal depression, as well as the perception of social support measured after the first postpartum trimester. In line with existing research, it is hypothesized that postnatal depression may impair emotional responsiveness, leading to weaker bonding, less positive interactions, and difficulty in addressing the infant’s needs. Conversely, the perception of social support is expected to promote more effective interactions between mother and child, supporting the child’s development by influencing specific aspects of the relational “dance steps”.

## 2. Materials and Methods

This study specifically examined the relational “Dance” during the neurodevelopmental follow-up visits scheduled by neonatal units to monitor the developmental progress of preterm infants, while also exploring how sample characteristics may influence the mutual “Dance steps” unfolding between the caregiver and their child from 6 to 9 months of corrected age. The study was conducted in a neonatal outpatient follow-up clinic, where the collaboration between neonatologists and pediatric psychologists was crucial. In this setting, caregivers received continuous support and guidance from both professionals in recognizing their infant’s developmental milestones at various follow-up stages. Additionally, they were supported in managing caregiving responsibilities, building scaffolding, and developing coping strategies, all of which contribute to fostering parenting competence.

### 2.1. Study Design

The present prospective observational study is part of a larger research project aimed at exploring the configuration of the caregiver–infant relationship during the child’s first year according to the Dance Steps model.

This study was conducted according to the Declaration of Helsinki—ethical principles for medical research involving human subjects; this study did not require ethics committee approval because it was part of the intervention protocol provided by the neonatal follow-up clinic as a corporate procedure.

### 2.2. Participants

A total of 30 mother–preterm infant dyads were enrolled in the study during follow-up visits scheduled by the neonatal intensive care unit (NICU) of “Villa Sofia-Cervello” Hospital. The caregivers, aged between 29 and 44 years (M = 35.83, SD = 4.27), were all of Italian nationality. Dyads with caregivers of different nationalities were excluded, as the caregiver–infant relational “Dance” cannot be considered culturally neutral, given the significant impact of cultural variables on caregiving practices [67,68,69]. For parents, the presence of chronic conditions, such as diabetes, cardiovascular disorders, and psychiatric disorders, represented an exclusion criterion. The infants (15 male; 15 female) were born moderately preterm (gestational age > 32 weeks, birth weight between 1500 and 2000 g) without significant developmental impairments attributable to preterm birth; additionally, all infants involved had a hospitalization period of 18–20 days. None of the infants involved in the sample presented specific health conditions during the follow-up period. As a result, dyads involving infants born very or extremely preterm, as well as those with preterm infants who had disabilities, deformities, or severe organic diseases, were excluded.

### 2.3. Procedure and Stages

Before initiating the monitoring of the relational “Dance” of the dyads at the two time points scheduled at 6 and 9 months of corrected age, an assessment was conducted at the end of the first trimester, coinciding with one of the neurodevelopmental follow-up visits at the reference hospital. This assessment aimed to evaluate the mothers’ perceived social support and any signs of postnatal depression.

Following this assessment, and up until the first 6-month time point, the dyads participated in a brief monthly program designed to support parenting competence. This program continued until the two interaction monitoring steps were carried out at 6 and 9 months of the child’s corrected age.

Regarding the two time points for monitoring the relational “Dance”, it is important to highlight that, at around 6 months of age, the child develops greater mobility and acquires new skills in communication, social interactions, play, feeding, and sleep. By 9 months, the child begins using the caregiver’s cues to make independent decisions, and parents face additional challenges in adapting to the child’s evolving motor and cognitive abilities [70,71,72].

Observations were conducted using video recordings of caregiver–infant interactions during the neonatal follow-up visits at 6 and 9 months of corrected age. Participating caregivers provided signed consent for the video recordings and agreed to the processing of sensitive data, including health information for both themselves and their infants, which was necessary for assessing the children’s developmental progress.

All video recordings took place within the hospital setting, typically lasting 3 to 5 min, and captured face-to-face interactions during unstructured play sessions. Caregivers were instructed to play freely with their child using a set of toys (ball, rattle, book, toy train, and stuffed animal) provided on a properly prepared mat. To accurately capture the mother–infant interactions, the camera was positioned to ensure simultaneous visibility of both participants, either side-by-side or in a way that both remained within the frame.

The observed mother–infant dyads had previously been involved in the outpatient neuropsychological follow-up program for preterm infants established by the Neonatology Unit at the “Villa Sofia-Cervello” Hospital in Palermo.

The observational tool was applied by a trained psychotherapist with extensive experience in Gestalt therapy, including at least 8 years of training and private practice, and a strong familiarity with the “Dance Steps” model. This professional had received specific training in using the observational instrument and was responsible for analyzing the video recordings to capture the nuanced dynamics of the relational “Dance”.

### 2.4. Measures

Sociodemographic variables were collected through a short questionnaire designed to collect information regarding maternal age, parity, maternal education level, marital and employment status, type of delivery, and the child’s sex.

The Multidimensional Scale of Perceived Social Support (MSPSS) was administered to identify the mothers’ perceived level of social support with family, friends, and significant others. The MSPSS is a 12-item questionnaire with a 7-point Likert scale and has been shown to be a reliable and valid measure of perceived social support. Scores between 12 and 35 indicate a low level of perceived support, scores between 36 and 60 highlight medium support, and scores between 61 and 84 suggest a high level of perceived support. It has been used in a variety of research studies, including studies of mental and physical health, coping, and social support [73,74].

The Edinburgh Postnatal Depression Scale (EPDS) is a validated self-report screening tool designed to support high-quality, evidence-based healthcare by assessing patients for depression. Healthcare professionals recommend EPDS screening during pregnancy and the postpartum period. The 10-item EPDS is user-friendly and has proven to be an effective screening tool. A score of 13 or higher on the EPDS indicates a positive screening for depression, suggesting that mothers with scores above this threshold are likely experiencing a depressive disorder with different degrees of severity [75,76].

The observation of the interactions was carried out through the “Dance step” grid, which is divided into 24 behaviors for the caregiver and 24 behaviors for the child and describes the relational dance in a mirror-like manner. Specifically, there are 3 items for each of the 8 dance steps included in the grid and described in Section 1.1:

A. Building together the sense of the ground; B. Perceiving one another; C. Acknowledging one another; D. Adjusting to one another; E. Taking bold steps together; F. Having fun; G. Connecting: This interaction fosters a sense of mutual accessibility and agency for both parties, enhancing their bond; H. Entrusting oneself to the other/Taking care of the other.

### 2.5. Statistical Analysis

Categorical variables were expressed as percentages (maternal age, educational level and employment status of the mothers, parity, marital status, type of delivery, the child’s sex, and dance steps frequencies), see Table 1. All clinical data regarding “Dance steps” were presented in terms of means and standard deviation (SD).

Given the constraint of a small sample size (30 dyads) and the frequency-based nature of our data, we selected non-parametric methods for the analyses. Normality testing revealed that the distribution of scores for the socio-demographic variables did not follow a normal distribution, further supporting our decision to use non-parametric methods, which are better suited for datasets that do not meet parametric assumptions. This approach enables us to accurately capture relational patterns in mother–infant interactions despite sample size limitations.

The following analyses were conducted:

- Descriptive analysis of relational “Dance” patterns activated by the mother and infant at 6 and 9 months of the infant’s corrected age.
- Non-parametric analysis was conducted to examine possible differences in “Dance steps” at 6 and 9 months of the infant’s corrected age for mother–infant dyads, using the Wilcoxon test.
- Rank-based analyses of variance were conducted to examine the mother’s and infant’s dance steps at 6 and 9 months of the infant’s corrected age in relation to socio-demographic variables using the Mann–Whitney U test for independent samples for maternal age, child’s sex, parity, and type of delivery and the Kruskal–Wallis test for maternal education and employment status. The effect size of the significant results was also measured by calculating specific indices (r of Rosenthal and eta-squared—η^2^).
- Co-occurrences between behavioral flows of the mother and the infant were explored using the correlation test of Spearman (ρ).
- Descriptive analysis of postnatal depression (EPDS) and analysis of variance (Mann–Whitney U test for independent samples) between maternal depression (presence or absence) and the mother’s and infant’s dance steps at 6 and 9 months of the infant’s corrected age.
- Descriptive analysis of perceived social support (MSPSS) and correlations (Spearman’s test, ρ) between the total and specific subscales of MSPSS and mothers’ and infants’ “Dance steps” at 6 and 9 months.

*p* < 0.05 was considered significant. Statistical analysis was performed by commercial software (IBM^®^ SPSS^®^ Statistics 28.0).

## 3. Results

### 3.1. Dance Steps Configuration at 6 and 9 Months

The relational dance configuration of the infant and mother at 6 months was characterized by the prevalence of certain steps (see Table 2). Specifically, with regard to infants, the steps “perceiving one another”, “building together the sense of the ground”, and “adjusting to one another” were observed as predominant (in reference to the mean) in the relational dance activated in the relationship with the mother; the step “taking bold steps together”, on the other hand, is scarcely present (χ^2^ 132.34, df 7; *p* < 0.001). Mothers, in dancing with their 6-month-old infant, also significantly activate mostly “perceiving one another”, followed by “building together the sense of the ground”; however, unlike infants, they also predominantly activate “taking bold steps together” (χ^2^ 144.81, df 7; *p* < 0.001).

Regarding the mother–infant relational dance at 9 months of the infant’s corrected age (Table 2), observational data showed that infants mainly activate the steps “perceiving one another”, “acknowledging one another”, and “adjusting to one another”; the step “entrusting oneself to the other/taking care of the other” is poorly represented (χ^2^ 131.96, df 7; *p* < 0.001). Mothers, in their relationship with 9-month-old infants, primarily activate the steps “perceiving one another”, “acknowledging one another”, “adjusting to one another “, and “taking bold steps together” (χ^2^ 124.08, df 7; *p* < 0.001).

### 3.2. Dance Step Trends at 6 and 9 Months

Regarding the relational dance trends between 6 and 9 months in relation to mothers and infants (Table 2), analysis of variance by ranks for two related samples (Wilcoxon’s test) showed, in infants, a statistically significant change in two steps, “acknowledging one another” (*p* = 0.001) and “taking bold steps together” (*p* < 0.001), as the means significantly increase at 9 months. The trend of the dance enacted by mothers shows statistically significant differences regarding several steps, with “building together the sense of the ground” (*p* = 0.002) and “perceiving one another” (*p* = 0.02) decreasing at 9 months. On the other hand, “acknowledging one another” (<0.001) and “connecting to one another” (<0.001) increased at 9 months.

### 3.3. Co-Occurrences in Dance Steps at 6 and 9 Months Between the Mother and Infant

The co-occurrences calculated in terms of correlation between the individual dance steps of mothers and infants at 6 and 9 months highlight some significant correlations, which differ according to the age of the child.

At 6 months, Spearman’s test correlations reveal several significant co-occurrences between mothers’ and infants’ behavioral patterns:

- Infants’ “building together the sense of the ground” dance step significantly correlates with mothers’ “building together the sense of the ground”, “adjusting to one another”, and “entrusting oneself to the other/Taking care of the other” dance steps.
- Infants’ “perceiving one another” step significantly correlates with mothers’ “perceiving one another”, “taking bold steps together”, and “having fun” steps.
- Infants’ “acknowledging one another” step correlates with mothers’ “perceiving one another”, “having fun”, and “entrusting oneself to the other/taking care of the other” steps.
- Infants’ “adjusting to one another” step correlates with mothers’ “perceiving one another”, “having fun”, and “connecting” steps.
- Infants’ “taking bold steps together” step correlates with mothers’ “building together the sense of the ground”, “adjusting to one another”, and “connecting” steps.
- Infants’ “having fun” step correlates with mothers’ “building together the sense of the ground”, “taking bold steps together”, and “connecting” steps.
- Infants’ “connecting” step correlates with mothers’ “building together the sense of the ground”, “taking bold steps together”, “having fun”, “connecting”, and “entrusting oneself to the other/taking care of the other” steps.
- Infants’ “entrusting oneself to the other/taking care of the other” step correlates with mothers’ “taking bold steps together” and “having fun” steps.
- These correlations are detailed in Table S1 (see Supplementary Materials).

At 9 months, Spearman’s correlation index showed the following statistically significant co-occurrences:

- Infants’ “building together the sense of the ground” step negatively correlates with mothers’ “entrusting oneself to the other/taking care of the other” step.
- Infants’ “perceiving one another” step correlates with mothers’ “having fun” step.
- Infants’ “acknowledging one another” step correlates with mothers’ “having fun” step.
- Infants’ “adjusting to one another” step correlates with mothers’ “adjusting to one another” and “having fun” steps.
- Infants’ “having fun” step correlates with mothers’ “having fun” step.
- Infants’ “entrusting oneself to the other/taking care of the other” step correlates with mothers’ “building together the sense of the ground”, “having fun”, and “entrusting oneself to the other/taking care of the other” steps.

These correlations are detailed in Table S2 (see Supplementary Materials).

### 3.4. Dance Steps at 6 and 9 Months Related to Sociodemographic Variables

An analysis of dance steps at 6 and 9 months was conducted with the Kruskal–Wallis and Mann–Whitney U tests in order to detect differences between the mother and child in relation to the sociodemographic variables considered (e.g., maternal age, child sex, parity, type of delivery, and the educational level and employment status of mothers). The results showed several statistically significant differences.

Regarding the influence of the maternal age variable on the relational dance between mothers and infants, the analysis revealed statistically significant differences in the mothers’ dance, especially at 9 months. Specifically, the steps “acknowledging one another”, “adjusting to one another”, and “connecting” are more prominent in the relational dance of the older mothers in the group (those aged 35–44 years). These mothers also engage in the step “entrusting oneself to the other/taking care of the other” more significantly at 6 months when compared to mothers aged 25–34 (Table S3 in Supplementary Materials).

Regarding the child’s sex, statistically significant differences are shown at 6 months in the “adjusting to one another” and “entrusting oneself to the other/taking care of the other” steps enacted by mothers with male children. At 9 months, the child’s sex appears to have a statistically significant effect on all the “dance steps” acted out by mothers, except for “perceiving one another” and “acknowledging one another” (Table S4 in Supplementary Materials). Again, mothers act out the steps more in the presence of a male child.

With reference to the infant’s “dance steps” at 6 and 9 months, analysis of variance in relation to the child’s sex variable highlights statistically significant differences at both 6 and 9 months relative to three steps (“adjusting to one another”, “connecting”, and “entrusting oneself to the other/taking care of the other”) that are more frequently acted by males. Furthermore, at 6 months, males also significantly perform the “building together the sense of the ground” step more than females (Table S5 in Supplementary Materials).

As regards the parity variable, statistically significant differences are again shown at 6 months in relation to the Dance steps “adjusting to one another” and “entrusting oneself to the other/taking care of the other”, which are most-performed by multiparous women. In contrast, this variable does not appear to be a discriminating factor at 9 months (Table S6 in Supplementary Materials).

In relation to the infant’s “dance” at both times, the parity variable appears significant only with respect to the step “perceiving one another” at 6 months. It would seem that children of multiparous mothers activate it significantly less than children of primiparous mothers.

Regarding the type of delivery, there is a significant tendency for mothers who had a cesarean delivery to activate “dance steps” to a greater extent than those who had a spontaneous delivery. In particular, at 6 months, this difference relates exclusively to the adapting and letting go steps, while at 9 months, the statistically significant difference relates to all “dance steps”, except “perceiving one another”, which still has a higher rank than in women who had a spontaneous delivery (Table S7 in Supplementary Materials).

With regard to possible differences in the infant’s “dance” when referring to the type of delivery variable, the analysis of variance shows a significant tendency at 6 months in the steps “building together the sense of the ground”, “acknowledging one another”, “adjusting to one another”, and “connecting” in babies born by spontaneous delivery to a lesser extent than in those born by cesarean section; we find the same tendency at 9 months regarding the steps “adjusting to one another”, “connecting”, and “having fun” (Table S8 in Supplementary Materials).

Concerning the mother’s educational level, at 6 months, all “dance steps” performed by mothers are activated in a significantly different way, with a tendency toward a lower presence of the “dance steps” in mothers with a lower educational background (middle school). Even at 9 months, the lower educational level seems to orient differences in the activation of almost all “dance steps”, except for the “perceiving one another” and “taking bold steps together” steps (Table S9 in Supplementary Materials).

With reference to the infant’s “dance steps” in relation to the maternal education variable, analysis of variance shows statistically significant differences at both 6 and 9 months relative to two steps (“having fun” and “entrusting oneself to the other/taking care of the other”) that are more frequently acted in infants with a mother who has a lower sociocultural level. The low educational level of the mother seems to direct further specific differences at 6 months, where infants tend to perform significantly fewer “building together the sense of the ground”, “taking bold steps together”, and “connecting” steps in the presence of low maternal education (Table S10 in Supplementary Materials).

Lastly, in relation to employment status, statistically significant differences in “dance steps” are mostly found at 6 months, relative to the steps “taking bold steps together” and “having fun”, which are acted out more by employed women (Table S11 in Supplementary Materials).

Further differences in infants’ “dance steps” in relation to the sociodemographic variables considered refer to a significantly lower presence of the “perceiving one another” step in 6-month-old infants with mothers who are not employed and are homemakers.

Marital status was not considered in the analysis of variance because the group consisted almost entirely of married or cohabiting women in stable relationships.

### 3.5. Dance Steps at 6 and 9 Months Related to Postnatal Depression and Perceived Social Support

As mentioned above, before monitoring of the mother–child relational “dance” was started, EPDS and MSPSS questionnaires were administered following the first trimester after delivery. The results on postnatal depressive conditions obtained through the EPDS showed that approximately 30% of the sample (nine subjects) had a score above the clinically significant cut-off (EPDS > 13). Regarding perceived social support, on the other hand, as measured through the MSPSS, a high level of perceived support (both total and in the subscales) was found in the entire sample (Table 3).

Analysis of variance revealed some important significant differences between the “dance steps” of mothers without depressive symptoms (EPDS < 13) and those of mothers with a clinically significant score (EPDS > 13). Specifically, the presence of depressive symptoms seems to negatively affect, especially at 6 months of the child’s corrected age, the frequency of the “perceiving one another”, ”having fun”, and “connecting” steps, while at 9 months, mothers with an EPDS score > 13 have a significantly lower frequency than that of the other mothers only in reference to the “having fun” step, although all “dance steps” appear less in mothers with postnatal depressive symptoms (Table S12 in Supplementary Materials).

On the other hand, regarding the possible effect of the mothers’ depressive symptoms on the children’s relational “dance”, the analysis of variance shows several significant differences at both 6 and 9 months, at the expense of children with mothers showing depressive symptoms. In fact, the latter at 6 months show a significantly lower frequency of the steps “acknowledging one another”, “adjusting to one another”, “taking bold steps together”, and “connecting”; at 9 months, all steps are significantly less activated than in the other children (mothers with EPDS < 13) (Table S13 in Supplementary Materials).

The correlation between individual dance steps of mothers at 6 months and perceived social support shows a significant inverse relation between the “connecting” step and the Total score and Friends subscale of MSPSS (Table S14 in Supplementary Materials).

At 9 months, the correlation between the mother’s “dance steps” and perceived social support shows a significant relation between the “acknowledging one another”, “taking bold steps together”, and “entrusting oneself to the other/taking care of the other” steps and the MSPSS Family subscale (Table S15 in Supplementary Materials).

Looking instead at the possible influence of the mothers’ perceived social support on the child’s relational “dance”, the data show only a significant impact at 6 months with respect to the MSPSS Total score concerning the step “entrusting oneself to the other/taking care of the other” (ρ = 0.39; *p* = 0.03).

## 4. Discussion

This study examines the configuration of early interactions between mothers and moderately preterm infants during the child’s first year according to the “dance steps” model—a phenomenological (focused on the experience as it unfolds in the immediate present), aesthetic (rooted in sensory perception), and field-oriented approach to understanding the reciprocal movements between the caregiver and infant in meaningful interactions [19]. Our research is framed within the existing literature, which has highlighted the significant impact of the quality of early mother–infant interactions on the development of preterm infants across all developmental domains [3,10,11]. Particularly during the first months of life, interactions with primary caregivers represent the most crucial resource for structuring the infant’s experiences [77].

In this study, according to the “dance steps” model, the specificity of the reading of early caregiver–child interactions focuses not on a separate observation of the individuals’ behaviors in the interaction but on their interactive reciprocity in a logic of dynamic co-regulation; the mother and child both contribute to the quality of the dyadic interaction [78].

In this regard, as pediatric psychology suggests, the “dance steps” of the reciprocity model can be seen as essential for ensuring the well-being of the neuropsychological developmental trajectory across various domains [79]. The primary domains addressed in the “dance steps” model include identity, emotions, and relationships [80,81,82,83], which are considered within their specific modular frameworks. Additionally, pediatric psychology views the experience of contact promoted by the “dance steps” as part of a higher-order strategic motivational system [84], oriented by the interplay between predisposition, phenomenological experience, and epigenetic contributions [19,85].

Dyadic interaction, understood as early reciprocity, can be significantly affected by risk conditions such as preterm birth. In fact, it is essential to deepen our understanding of early mother–infant interactions under preterm birth conditions to identify effective intervention pathways and best practices that can promote the developmental well-being of both the child and the mother–child relationship.

The specificity of the “dance steps” model, and its associated observational tool, should be highlighted as it allows for the detailed analysis of reciprocal movements in mother–child interactions. This model captures the co-occurrence of behavioral flows, emphasizing the contribution of both the mother and child to each interactional exchange.

Moreover, this study aimed to analyze how the relational mother–infant “dance” evolves from 6 to 9 months of corrected age, because early caregiver–child interactions are highly dynamic during the first year of life, influenced by the child’s rapid maturational changes and the caregiver’s unique contextual and dispositional characteristics. The choice of these two developmental time points (6 and 9 months) is based on their significance for early development, as they correspond to critical stages in cognitive and relational learning—particularly for preterm infants [70,71,72].

Finally, this study examines the impact of specific sociodemographic (e.g., the child’s sex, maternal education level, employment status, parity, and mode of delivery) and maternal psychological factors (e.g., postnatal depression and perceived social support) on the quality of the relational “Dance”. This approach aims to determine how these variables either facilitate or hinder the expected activation patterns for each “dance step”.

### 4.1. Trends of the Relational “Dance” Between Mothers and Moderately Preterm Infants from 6 to 9 Months of Age

The results concerning the configuration of the mother–infant relational dance at 6 and 9 months, as well as the trend observed across these two developmental stages, highlight certain features of reciprocity. For many dance steps, these characteristics appear consistent across both time points, reflecting synchrony and attunement between mother and child during their interactions.

These features primarily involve the prevalence of interactive patterns centered around “perceiving one another” (the most frequently enacted step by both mothers and infants at 6 and 9 months), which is linked to the shared sensory experience of being together. Another prominent pattern is “building together the sense of the ground” (the second most frequent step primarily at 6 months), which refers to the establishment of a clear relational context that both participants inhabit. This step also involves seeking physical proximity to the other. Additionally, the pattern of “adjusting to one another” emerged as one of the most commonly enacted behaviors by both parties across both time points.

The dominance of these steps suggests a mutual need for closeness, within a shared space where intuitive understanding occurs. This need for the presence of the other—seen as a necessary backdrop from which to move—aligns with the child’s developmental trajectory, particularly from 6 months onward, as they begin to reach important developmental milestones. For example, this phase is marked by an increased curiosity towards the environment (early precursors of agency) [86], supported by the child’s advancing motor and verbal abilities (e.g., the onset of babbling). The mother, in turn, by activating these steps meaningfully, seems to show her intention to adapt to the child’s new competencies while remaining a reachable and responsive physical reference point.

The step “acknowledging one another” is also present across both stages of the dyadic dance, becoming particularly prominent at 9 months. It should be noted that this is a period when the child’s communicative and relational skills have significantly increased compared to the previous three months. This shift enhances the aesthetics of dyadic interaction, expressed through mutual recognition of each other’s intentions and intentionality. For instance, the mother and child seek each other’s gaze, facial expressions change in response to one another, and verbal interactions become more frequent, in a dynamic of reciprocity where the other becomes the focal point of shared interest [87]. This process supports the development of the child’s internal representation of the mother as a subject with whom experiences can be shared.

Faced with this change in the child’s engagement, the mother tends to adjust by focusing more on acknowledging his/her new abilities, decreasing her active role in introducing new variables into the interaction—unlike what was observed at 6 months.

When analyzing the unique characteristics of certain steps in the mother–infant relational dance, it becomes evident that at 6 months, the step “taking bold steps together” is predominantly enacted by the mothers, with limited engagement from the infants. This suggests that the mothers are attempting to introduce environmental variables into the interaction (such as toys and verbal stimuli) to elicit a response from the child, who is perceived as less responsive. Therefore, through this strategy, the mothers seem to encourage the infant to become more active and engaged in the relationship. On the other hand, at this age, infants are not yet able to actively position themselves within the relationship; consistent with their developmental stage, behaviors that introduce novelty into the interaction are quite limited, as their capacity to grasp the intention of the other will only emerge around 9 months [86]. This phenomenon is even more pronounced in the case of preterm births, where infants often show lower alertness and, concurrently, caregivers tend to increase their level of stimulation [9,12]. Studies indicate that caregivers of preterm infants often find it difficult to tolerate the slower behavioral responses of their infants, leading them to be more intrusive, active, and stimulating, particularly in the early months of life where preterm infants appear less responsive and attentive compared to full-term infants [4,9,11,12,40].

This situation poses a risk to maternal parenting competence when mothers fail to recognize the specific developmental characteristics of preterm infants and instead make comparisons with full-term children, which can result in significant biases [88,89,90]. However, some researchers [40,91] have contested these findings, arguing that interaction patterns characterized by high levels of stimulation could be beneficial for preterm infants, compensating for their limited communicative abilities.

A further reflection should be made regarding the steps associated with the ability of both the mother and child to relax while being together, such as “having fun”, “connecting”, and “entrusting oneself to the other/taking care of the other”, through physical proximity (e.g., touching or gazing), which are notably absent in the relational dance, particularly at both 6 and 9 months. This pattern suggests a potential risk for the quality of the relational dance, as the mother’s perception of the child as unresponsive may lead her to focus excessively on stimulating the infant, sacrificing opportunities for relaxation and shared enjoyment. This excessive focus on stimulation keeps the mother in a state of tension and heightened alertness, preoccupied with “having to do it herself”, and results in the activation of controlling interaction patterns [88,92]. In fact, as the results will later show through the analysis of co-occurrences, moments of relaxation can support the infant’s ability to relax as well.

### 4.2. The Reciprocity of the Relational “Dance”: Co-Occurrences

If the trend in the configuration of the mother–infant interaction highlights predominant qualitative characteristics in the relational dynamic of our sample, analyzing the co-occurrences between the two behavioral flows helps identify potential positive associations between maternal and infant behaviors. These insights can suggest effective strategies to support parental competence.

In line with the predominant patterns observed in mothers and infants, the co-occurrences reveal reciprocity at 6 months in steps such as “building together the sense of the ground”, “perceiving one another”, and “connecting”. In particular, the “building together the sense of the ground” step remains prevalent in both partners of the interaction even at 9 months.

This suggests that intimacy and mutual understanding at such an early age are largely influenced by a secure relational context in which the caregiver actively seeks and recognizes the infant, adapting to his/her needs and acknowledging his/her developmental resources and vulnerabilities. The “dance steps” model thus suggests that constructing a shared relational background is an essential condition for initiating a well-balanced relational dance in the early months of life. Therefore, where deficient, promoting and enhancing behaviors that contribute to building a shared relational ground in the first months of life is crucial, as it shapes the understanding of the presence of the other and the relational context itself.

It is particularly interesting how, at 6 months, the maternal behaviors of “having fun”, “connecting”, and “entrusting oneself to the other/taking care of the other” are associated with most of the infant’s steps. In fact, they are linked both to the most frequent steps of the child at this age—such as “building together the sense of the ground”, “perceiving one another”, and “acknowledging one another”—as well as to those less frequent at 6 months, such as “taking bold steps together” and “connecting” (as shown in the trend pattern description, see Section 4.1). This finding suggests that the mother’s relaxation and playfulness during interactions encourage the child to display more complex and proactive behaviors. This significant association was also observed at 9 months.

Therefore, while supporting reciprocity in creating a shared experiential ground is essential, it is equally crucial to foster mothers’ ability to relax and have fun in their interactions, helping them recognize that these modes are beneficial for their child’s development, particularly in promoting the child’s agency and understanding of the other’s intentions but also as a chance to bring novel elements to the interaction.

Interestingly, the child’s step “entrusting oneself to the other/taking care of the other” is significantly associated not with a mother performing the same step but with a mother engaged in playful behaviors and novelty introduction. This suggests that the child may feel encouraged to trust and let go when perceiving a mother who is relaxed and enjoying herself.

One behavior that warrants further consideration is the infant’s “taking bold steps together”. While this was one of the least frequent behaviors in the infant’s relational dance at 6 months (as previously noted in Section 4.1), the co-occurrence analysis reveals its significant association with maternal behaviors that not only maintain the relational ground and adapt to him but also actively connect with him. This involves more than simply perceiving the infant: it entails acknowledging and adapting to the infant’s evolving characteristics, particularly through physical contact. The correlation suggests that the activation of this connection mode can encourage the infant, as early on as at 6 months of age, to display more active behaviors.

The infant’s “acknowledging one another” step (where s/he actively engages with the mother to share interactive experiences) is also significantly associated with maternal behaviors involving seeking out the child and having fun in the interaction. Thus, supporting mothers in adopting these behaviors can promote the child’s development of agency and autonomy, which will become more evident in subsequent months.

Finally, at 9 months, one negative correlation emerges between the infant’s “acknowledging one another” and the mother’s “building together the sense of the ground”. Within the framework of the interaction model, this negative association suggests that if the child shows a lack of availability or difficulty in recognizing the other and, consequently, himself in that relational context, the mother tends to enhance behaviors that foster a sense of shared belonging. The prominence of this maternal behavior can be seen not as an attempt to control but rather as an effort to draw the child closer and strengthen mutual closeness, which, as previously discussed, is crucial for the well-being of both the mother and infant.

### 4.3. Impact of Sociodemographic Variables, Postnatal Depression Symptoms, and Perception of Social Support on “Dance Steps” at 6 and 9 Months of Age

This study also explored potential differences in the configuration of mother–infant interaction patterns at 6 and 9 months, considering the impact of various sociodemographic variables related to the mother and infant as well as the potential effects of postnatal depression symptoms and perceived social support measured at the end of the first postpartum trimester.

Specifically, maternal education, employment status, parity, type of delivery, the child’s sex, and maternal age were considered. Previous research has shown that sociodemographic factors significantly influence early interactions between preterm infants and caregivers, especially for mothers experiencing psychological stress [35,36].

The effect of maternal age is limited to only a few specific steps in the mother’s relational dance; these differences are mainly linked to the mother’s tendency to seek closeness with the child, both in terms of perceptual recognition and in adapting to the child’s needs. Research on the impact of maternal age is often dated and presents mixed findings. Our findings align with studies suggesting that older mothers tend to engage more positively in interactions with their infants, create a more nurturing home environment [93], and act as more discerning observers, detecting aspects of their child’s behavior that younger mothers may overlook [94]. It is likely that the maturity associated with age encourages mothers to focus on tuning in to their child, responding to the uniqueness of the child’s needs while also fostering opportunities for mutual connection.

Regarding the variable of the child’s gender, our results show a richer reciprocal interaction in terms of the frequency of behaviors with boys. At 9 months, when the child’s developmental competencies increase, mothers become more engaged with boys, creating a recognizable relational context, enjoying time together, connecting, and engaging in physical touch. Boys, in turn, show more physical closeness and adjust more to the interaction, considering the mother’s specific cues.

In addition, these results require further exploration, as the literature on mother–preterm infant interactions and the infant’s sex is limited and often contradictory. For example, our findings align with studies indicating greater challenges for mothers of daughters [45], while others have found no significant differences in maternal stimulation based on the child’s gender [49]. However, our results differ from studies that suggest that attachment difficulties are more common with boys [47,49], as well as higher levels of parental stress and fear [35]. On the contrary, several studies have noted higher parental sensitivity towards daughters [36].

One key variable shaping the relational dance at 6 and 9 months is the mothers’ socio-cultural context, which was assessed through educational level and employment status. Higher maternal education was associated with greater activation of all interactional steps, particularly at 6 months. In turn, employment status impacted specific steps, such as “taking bold steps together” and “having fun” at 6 months and “acknowledging one another” at 9 months. These steps were less frequently displayed by unemployed mothers.

The children’s behaviors at 6 months were also influenced by the mother’s educational background and employment, although to a lesser extent. Infants of mothers with higher education tended to be more active in interactions, initiating novel behaviors and engaging in playful contact with the caregiver. This also corresponded with a greater tendency to approach the caregiver and establish a shared sense of presence and belonging.

Regarding maternal employment status, it is notable that 6-month-old infants showed significantly lower engagement in the “acknowledging one another” step when their mother was unemployed.

These findings, which indicate a richer relational dance in mothers with higher education levels and, correspondingly, in their children, are consistent with research highlighting the impact of maternal socioeconomic and cultural factors as predictors of maternal sensitivity and cooperative behaviors in preterm infants [95,96], as well as more positive home environments [35,37]. Conversely, mothers with lower education levels have been associated with more negative control over their children [36].

A mother’s educational level may shape her perceptions and representations of her child, suggesting that mothers with higher education have greater cognitive resources to recognize their child’s developmental characteristics, including strengths and vulnerabilities linked to preterm birth. This aspect may allow them to perceive the child as more manageable, facilitating better attunement to his/her needs. In turn, it positively influences the child’s interactive patterns, in line with studies showing that children of mothers with higher education are less irritable [38,61] and engage in more positive interactions with increased vocalizations and smiling [97]. However, the impact of this variable warrants further investigation in future studies, considering the limited number of available studies and occasionally contradictory results.

Regarding the mode of delivery (spontaneous/cesarean), our study shows that it may influence specific differences in the relational dance of both mothers and children at 6 and 9 months. Specifically, cesarean delivery appears to be associated with a higher frequency, especially at 9 months, of steps such as creating a sense of ground, adapting to the child, and playing or having fun together. Similarly, children born via cesarean seem more engaged and active in the interaction with their mothers, showing greater responsiveness and playfulness compared to those born vaginally. This observation contrasts with other studies indicating that cesarean-born infants may exhibit greater irritability, leading to more challenging interactions with their caregivers [35].

Our findings thus suggest that the experience of cesarean delivery may be perceived as less stressful compared to natural childbirth, fostering a more relaxed interaction style for mothers. This is consistent with studies such as the one by Monti et al. [46], which found that cesarean birth has a protective psychological impact, being less associated with negative postpartum experiences such as excessive worries and phobic anxiety disorders. Similarly, lower stress axis activation was found in cesarean mothers [98], while no significant association between postpartum depression and cesarean delivery was reported up to 16 months after birth [99]. However, these results contrast with other research [100], which indicated that cesarean delivery might increase the risk of postpartum depression.

Certainly, our findings call for further investigation, particularly because research on the relationship between birth type and the quality of the mother–infant relationship remains sparse and yields mixed results [101,102,103].

As for parity, our results indicate that it influences the mother–infant relational dance primarily only at 6 months. Multiparous mothers tend to adapt more to their children and engage in physical contact more easily, suggesting that greater experience with other children fosters a more synchronized and confident interaction. However, the children of multiparous mothers are less engaged in shared sensory experiences (“perceiving one another”) and these findings align with previous studies showing that primiparous mothers exhibit greater maternal responsiveness [104] and positive involvement, which correlates with more vocalizations from their infants [36].

Regarding the potential impact of maternal depressive symptoms on the quality of the relational dance during the early postpartum period, our results confirm the negative influence of this variable, even several months after birth. In fact, the assessment of postnatal depression was conducted at the end of the first trimester. Although few studies focus on maternal depressive symptoms in conditions of preterm birth within the first year post-discharge from NICU [105], available data point out that depressive symptoms tend to persist longer in mothers of preterm infants compared to those of full-term infants [89,106]. Furthermore, although the prevalence of postpartum depression risk typically decreases over time [105], in the case of preterm births, mothers often experience depressive symptoms for an extended period after delivery [107]. This prolonged impact may explain the difficulty these mothers have in connecting and enjoying time with their infants. Notably, the quality of the relational dance shows that mothers with depressive symptoms, at both 6 and 9 months, struggled more than others to connect with their infants and enjoy time together. This finding aligns with evidence showing that mothers with depressive symptoms tend to engage less in play and communication with their infants [108,109]. Additionally, our data reveal a significant impact of the mother’s emotional state on the child’s interactive behaviors. Children of mothers with depressive symptoms exhibited fewer dance steps compared to others, with this effect becoming more pronounced at 9 months, a crucial time for developmental milestones. At this stage, maternal depressive signs seem to discourage the child’s attempts at agency. It has been noted that mothers experiencing psychological distress often feel less involved in the relationship, which can negatively affect the security of the attachment bond [110].

Regarding perceived social support, we recall that the mothers in our sample appeared to be sufficiently supported mainly by family, and the literature confirms that instrumental support from primary group members (i.e., family, friends, and/or a significant other) is particularly effective in alleviating stress, as it reduces situational demands and conveys care and esteem [111].

Correlational analyses indicate that perceived social support has a specific and significant impact on certain “dance steps” enacted by mothers and infants at 6 and 9 months, suggesting important insights into the long-term effects of perceived support. Rather than a broad influence across all interaction patterns, this effect is seen in the mother’s increased engagement in physical and sensory connection with her child, particularly in managing physical closeness, especially at 9 months. Mothers with higher perceived support tend to interact with more physical connection, and children in these dyads appear more relaxed and confident in the caregiver relationship, an essential condition for the child’s developmental well-being.

Feeling supported, especially by family in the early postpartum months, seems to boost maternal confidence in interactions with more active and competent infants. This seems to allow mothers to engage more in recognition behaviors, making the interaction more interesting by introducing elements of novelty and relaxing into physical closeness with their child.

Our findings align with studies showing that informal support from family and friends significantly enhances postpartum women’s well-being [112], with a positive impact on perinatal mental health [113,114].

Research increasingly emphasizes the value of family-centered care in NICUs [115]. In particular, we would like to underline the importance of involving some significant figures (partners and/or family of origin) in the support pathways for mothers in the NICU, as mothers will especially need their assistance during the first year after NICU discharge; therefore, this involvement may be useful in sustaining perceived maternal competence [88,105].

### 4.4. Limitations and Future Directions

The present study, while providing valuable insights into mother–infant interactions through the analysis of “dance steps”, has certain limitations. Firstly, the small sample size limits the generalizability of the findings and does not allow for an in-depth exploration of potential individual or contextual factors. The sample was sourced from a single neonatal follow-up unit and predominantly included participants from stable relationships, limiting the diversity of sociodemographic variables. This uniformity in family structure may reduce the applicability of the findings to a broader range of family contexts. Moreover, the exclusion of fathers prevents a comparative analysis of maternal and paternal roles in early relational interactions. This is especially relevant considering the crucial role that fathers play in synchrony and regulatory processes in infant development.

To overcome these limitations, future research directions will include an increase in sample size, with a larger number of mother–infant dyads, as well as the inclusion of fathers. This will allow a more comprehensive examination of parental roles and whether the “dance steps” vary according to the presence of each caregiver. Furthermore, it is essential to further validate the observational model used in this study, with the aim of establishing specific developmental cut-offs to identify levels of synchrony and asynchrony between interactive steps. Defining these criteria would enable the tool to be used for the early detection of potential interaction difficulties.

In this context, the research project has already been extended to include a control group of dyads with full-term infants, to compare their interactive characteristics with those of preterm infants. This comparison is a critical step in validating the model and understanding whether the observed relational dynamics are attributable to the unique characteristics of preterm infants or reflect more general developmental factors.

## 5. Conclusions

The findings of our study highlight the dynamic nature of the mother–child relational dance, considering the children’s particularly rich and rapid developmental progress during the first year of life. A functional “dance” for the child’s growth should involve the synchrony of some steps and the asynchrony of others between the mother’s and child’s interactive flow. For instance, steps such as “building together the sense of the ground” or “acknowledging one another” must occur in synchrony at both 6 and 9 months, as they form a fundamental foundation for interactive reciprocity. Without a shared relational ground, it becomes difficult to introduce novelty, relax, or enjoy the interaction.

As the child’s skills develop, it is crucial for the mother to reduce her active role in proposing stimuli and allow the child to take a more active part in the interaction, fostering an alternation between figure and background. This synchrony and asynchrony in “dance steps” emphasize the need to support mothers in recognizing their child’s developmental needs after admission to the NICU, helping them to adjust their interactions to promote specific relational patterns. Therefore, it may be particularly important to include a segment of work on the specificity of the relational dance in the pathways of accompanying and supporting parents during the newborn’s NICU hospitalization. This means accompanying each pair of parents in reflecting on their relational “dance” with their newborn, considering the child’s developmental characteristics, and the specificity of their approach with their preterm infant, in order to make any changes to their own interactional patterns.

Furthermore, our data highlight the importance of mothers being able to relax during interactions with their preterm infants, as this facilitates mutual enjoyment and nurturing physical contact for development. Therefore, parenting skills support programs should also include experiential interventions that encourage caregivers to engage with their children in a relaxed manner, avoiding constant stimulation. This would encourage more active and structured reciprocal behaviors by the child.

The “dance steps” model thus seems to redefine caregiver sensitivity as not only responding to the child’s immediate needs but also addressing the need to relax and enjoy the interaction. This approach helps the child develop an early understanding of intimate co-regulatory relationships that are based on the enjoyment of being together.

In addition, support and accompaniment programs should consider the weight that certain sociodemographic variables have in guiding early mother–child relationships by tailoring interventions (e.g., modulating the intervention according to the mother’s educational level).

Similarly, support and accompaniment programs should include routine screening for maternal depressive symptoms, not only during hospitalization in the NICU but also in early neonatal follow-ups. Such screening is in fact a key preventive action [116], not only to provide timely support to the mother but also and especially to ensure the child’s fundamental right to experience healthy relationships, in the sense of being aligned with his/her developmental needs.

## Figures and Tables

**Table 1:** Sample sociodemographic description.

	Percentage
Maternal Age	%
25–34	40.0
35–44	60.0
Parity	
Primiparas	56.7
Multiparas	43.3
Level of Education	
Middle school	20.0
High school	33.3
Professional diploma	10.0
Degree	36.7
Marital status	
Married	80.0
Separated	10.0
Cohabiting	10.0
Employment status	
Employed	20.0
Seeking employment	53.3
Housewives	26.7
Delivery	
Spontaneous	36.7
C-section	63.3
Child sex	
Male	50.0
Female	50.0

**Table 2:** Relational dance for mothers and infants at 6 and 9 months of the infant’s correct age.

Dance Steps		6 Months	9 Months	Wilcoxon Test	
		Mean (SD)	Mean (SD)	Z	*p*-Value
Building together the sense of the ground	Mothers	9.6 (2.7)	8.2 (2.8)	−3.1	0.002 *
	Infants	5.9 (2.7)	5.2 (2.0)	−1.5	0.147
Perceiving one another	Mothers	15.7 (4.3)	13.8 (6.1)	−2.3	0.024 *
	Infants	6.2 (4.4)	7.5 (4.2)	−1.8	0.071
Acknowledging one another	Mothers	6.0 (2.7)	9.8 (5.0)	−4.0	0.000 *
	Infants	4.6 (2.1)	7.0 (4.1)	−3.3	0.001 *
Adjusting to one another	Mothers	7.7 (2.9)	8.6 (3.1)	−1.9	0.060
	Infants	5.6 (3.4)	6.6 (4.8)	−1.6	0.099
Taking bold steps together	Mothers	9.2 (3.1)	8.6 (3.5)	−0.6	0.567
	Infants	0.3 (0.6)	1.9 (2.1)	−3.5	0.000 *
Having fun	Mothers	5.8 (4.7)	5.0 (2.8)	−0.8	0.406
	Infants	2.9 (4.6)	3.8 (4.1)	−1.3	0.196
Connecting	Mothers	4.8 (3.9)	5.1 (3.6)	−4.0	0.000 *
	Infants	1.2 (2.2)	2.0 (2.2)	−2.0	0.050
Entrusting oneself to the other/ Taking care of the other	Mothers	3.1 (2.5)	2.5 (3.0)	−1.4	0.153
	Infants	1.3 (1.6)	1.0 (1.9)	−1.1	0.291

* *p* < 0.05.

**Table 3:** Descriptive Statistics of EPDS and MSPSS Scores.

		Mean (SD)
EPDS		6.9 (6.7)
MSPSS	Family	26.0 (2.7)
	Friends	23.4 (3.8)
	Significant others	24.7 (2.5)
	Total	74.1 (8.2)

Data are expressed as means (and standard deviations—SD—in parentheses). MSPSS. Multidimensional Scale of Perceived Social Support. EPDS. Edinburgh Postnatal Depression Scale.

## Data Availability

The original contributions presented in the study are included in the article, further inquiries can be directed to the corresponding author.

## Supplementary Materials

The following supporting information can be downloaded at: https://www.mdpi.com/article/10.3390/healthcare12222231/s1, Table S1: Co-occurrences in dance steps at 6 months between mother and infant; Table S2: Co-occurrences in dance steps at 9 months between mother and infant; Table S3: Differences in mother’s dance steps at 6 and 9 months by maternal age; Table S4: Differences in mother’s dance steps at 6 and 9 months by child sex; Table S5: Differences in infant’s dance steps at 6 and 9 months by child sex; Table S6: Differences in mother’s dance steps at 6 and 9 months by parity; Table S7: Differences in mother’s dance steps at 6 and 9 months by type of delivery; Table S8: Differences in infant’s dance steps at 6 and 9 months by type of delivery; Table S9: Differences in mother’s dance steps at 6 and 9 months by level of education; Table S10: Differences in infant’s dance steps at 6 and 9 months by level of education; Table S11: Differences in mother’s dance steps at 6 and 9 months by employment status; Table S12: Differences in mother’s dance steps at 6 and 9 months by postnatal depression; Table S13: Differences in infant’s dance steps at 6 and 9 months by postnatal depression; Table S14: Differences in mother’s dance steps at 6 months by perceived social support; Table S15: Differences in mother’s dance steps at 9 months by perceived social support.
