# Supplementary material for: Relational “Dance” Between Mother and Moderately Preterm Infant at 6 and 9 Months of Correct Age: Possible Risk and Protective Factors
Source: Healthcare (Basel). 2024 Nov 8;12(22):2231. doi: 10.3390/healthcare12222231 (PMC11593660; doi:10.3390/healthcare12222231)
Supplement: Supplementary file 1 [file healthcare-12-02231-s001.zip › healthcare-3289932-supplementary.pdf]

Table S1. Co-occurrences in dance steps at 6 months between mother and infant.

			Child's Dance steps							
			A	B	C	D	E	F	G	H
Mother's Dance steps	A	Spearman's correlation	.448*	-.051	.292	.309	.517**	.440*	.362*	.293
		Sign. (two-tailed)	.013	.791	.117	.097	.003	.015	.049	.116
	B	Spearman's correlation	.096	.382*	.439*	.389*	.113	.042	.303	.250
		Sign. (two-tailed)	.614	.037	.015	.034	.554	.827	.104	.182
	C	Spearman's correlation	.043	.185	.186	.129	.155	.074	.108	.214
		Sign. (two-tailed)	.823	.328	.326	.498	.413	.698	.569	.256
	D	Spearman's correlation	.424*	-.080	.209	.149	.375	-.094	.184	.094
		Sign. (two-tailed)	.020	.675	.268	.432	.041	.620	.331	.622
	E	Spearman's correlation	-.001	.380*	.268	.320	.169	.542**	.372	.399*
		Sign. (two-tailed)	.995	.038	.153	.084	.371	.002	.043	.029
	F	Spearman's correlation	.351	.382*	.379*	.446*	.354	.341	.475**	.580**
		Sign. (two-tailed)	.057	.037	.039	.014	.055	.066	.008	.001
	G	Spearman's correlation	.174	-.014	.301	.617**	.550**	.527**	.549**	.103
		Sign. (two-tailed)	.358	.943	.106	.000	.002	.003	.002	.587
	H	Spearman's correlation	.644**	-.303	.369*	.427*	.322	.126	.418*	.307
		Sign. (two-tailed)	.000	.104	.045	.019	.082	.506	.021	.099

*. The correlation is significant at the 0.05 level (two-tailed), **. The correlation is significant at the 0.01 level (two-tailed).

A. Building together the sense of the ground; B. Perceiving one another; C. Acknowledging one another; D. Adjusting to one another; E. Taking bold steps together; F. Having fun; G. Connecting; H. Entrusting oneself to the other/Taking care of the other.

Table S2. Co-occurrences in dance steps at 9 months between mother and infant.

Mother's Dance steps			Child's Dance steps A	B	C	D	E	F	G	H
	A	Spearman's correlation	-.047	-.276	-.490**	-.083	-.047	.327	-.029	-.003
		Sign. (two-tailed)	.806	.140	.006	.661	.803	.078	.877	.989
	B	Spearman's correlation	-.189	.027	-.041	.252	.008	.403*	-.069	-.242
		Sign. (two-tailed)	.318	.888	.831	.180	.965	.027	.716	.198
	C	Spearman's correlation	-.107	.149	.085	.301	-.024	.434*	-.002	-.310
		Sign. (two-tailed)	.573	.431	.657	.106	.901	.016	.992	.095
	D	Spearman's correlation	.055	.339	.307	.516**	.184	.553**	.327	-.117
		Sign. (two-tailed)	.772	.067	.099	.004	.330	.002	.078	.538
	E	Spearman's correlation	-.266	.049	-.100	.209	-.160	.215	.160	-.320
		Sign. (two-tailed)	.156	.799	.599	.268	.398	.255	.399	.085
	F	Spearman's correlation	.235	.017	.134	.412*	.013	.691**	.285	.107
		Sign. (two-tailed)	.212	.931	.481	.024	.944	.000	.126	.574
	G	Spearman's correlation	.031	-.073	-.217	.113	-.001	.484**	.466**	.029
		Sign. (two-tailed)	.870	.703	.250	.550	.997	.007	.010	.880
	H	Spearman's correlation	.644**	-.303	.369*	.427*	.322	.126	.418*	.307
		Sign. (two-tailed)	.000	.104	.045	.019	.082	.506	.021	.099

*. The correlation is significant at the 0.05 level (two-tailed), **. The correlation is significant at the 0.01 level (two-tailed).

A. Building together the sense of the ground; B. Perceiving one another; C. Acknowledging one another; D. Adjusting to one another; E. Taking bold steps together; F. Having fun; G. Connecting; H. Entrusting oneself to the other/Taking care of the other.

Table S3. Differences in the mother's dance steps at 6 and 9 months by maternal age.

Dance steps	Maternal age	6 months	Mann–Whitney test		r (ES)	9 months	Mann–Whitney test		r (ES)
		Mean rank	U	p		Mean rank	U	p	
Building together the sense of the ground	25-34	11.9	64.5	.117		15.0	99.0	.999	
	35-48	16.9				15.0			
Perceiving one another	25-34	14.4	92.5	.769		14.4	92.5	.774	
	35-48	15.4				15.4			
Acknowledging one another	25-34	12.5	71.5	.210		9.7	41.0	.008*	0.52
	35-48	16.5				18.2			
Adjusting to one another	25-34	13.8	86.0	.552		10.6	50.0	.028*	0.45
	35-48	15.7				17.7			
Taking bold steps together	25-34	12.1	67.0	.148		16.2	85.5	.550	
	35-48	16.8				13.0			
Having fun	25-34	14.0	87.5	.599		12.9	76.5	.317	
	35-48	15.6				16.3			
Connecting	25-34	12.8	75.0	.278		11.0	54.5	.044*	0.41
	35-48	16.3				17.5			
Entrusting oneself to the other/ Taking care of the other	25-34	11.1	55.5	.047*	0.41	13.3	80.0	.412	
	35-48	17.4				16.1			

* p < .05

r: r of Rosenthal; ES: Effect Size

Table S4. Differences in the mother's dance steps at 6 and 9 months by child sex.

Dance steps	Child sex	6 months	Mann–Whitney test		r (ES)	9 months	Mann–Whitney test		r (ES)
		Mean rank	U	p		Mean rank	U	p	
Building together the sense of the ground	Male	17.7	79	.160		20.6	35.5	.001*	0.58
	Female	13.3				10.4			
Perceiving one another	Male	15.2	108.5	.867		18.2	71.5	.087	
	Female	15.8				12.8			
Acknowledging one another	Male	15.0	105	.752		17.9	76.5	.132	
	Female	16.0				13.1			
Adjusting to one another	Male	19.5	52	.011*	0.46	19.4	54.5	.015*	0.44
	Female	11.5				11.6			
Taking bold steps together	Male	14.8	102	.661		21.4	23.5	<.001*	0.67
	Female	16.2				9.6			
Having fun	Male	17.1	88.5	.312		20.8	33	.001*	0.60
	Female	13.9				10.2			
Connecting	Male	17.4	84	.234		20.3	41	.003*	0.54
	Female	13.6				10.7			
Entrusting oneself to the other/ Taking care of the other	Male	19.5	52.5	.012*	0.46	18.7	64.5	.037*	0.36
	Female	11.5				12.3			

* p < .05
r: r of Rosenthal; ES: Effect Size

Table S5. Differences in the infant's dance steps at 6 and 9 months by child's sex.

Dance steps	Child sex	6 months	Mann–Whitney test		r (ES)	9 months	Mann–Whitney test		r (ES)
		Mean rank	U	p		Mean rank	U	p	
Building together the sense of the ground	Male	20.1	43	.003*	0.53	17.1	88	.281	
	Female	18.9				13.9			
Perceiving one another	Male	14.0	90	.347		15.7	109.5	.900	
	Female	17.0				15.3			
Acknowledging one another	Male	16.7	95	.461		15.6	111	.950	
	Female	14.3				15.4			
Adjusting to one another	Male	19.3	55.5	.017*	0.43	19.1	59	.026*	0.41
	Female	11.7				11.9			
Taking bold steps together	Male	17.4	84.5	.133		15.9	106	.782	
	Female	13.6				15.1			
Having fun	Male	16.6	96	.473		18.0	75	.112	
	Female	14.4				13.0			
Connecting	Male	19.3	55.5	.007*	0.43	18.7	64	.037*	0.37
	Female	11.7				12.3			
Entrusting oneself to the other/ Taking care of the other	Male	19.5	55.5	.007*	0.43	18.7	64.5	.010*	0.36
	Female	11.5				12.3			

* p < .05
r: r of Rosenthal; ES: Effect Size

Table S6. Differences in the mother's dance steps at 6 and 9 months by parity.

Dance steps	Parity	6 months	Mann–Whitney test		r (ES)	9 months	Mann–Whitney test	
		Mean rank	U	p		Mean rank	U	p
Building together the sense of the ground	Primiparas	12.9	66.5	.063		13.9	84	.256
	Multiparas	18.9				17.5		
Perceiving one another	Primiparas	16.5	93.5	.473		13.1	70	.088
	Multiparas	14.2				18.6		
Acknowledging one another	Primiparas	15.6	109.5	.966		15.9	103.5	.768
	Multiparas	15.4				15.0		
Adjusting to one another	Primiparas	12.2	55	.018*	0.42	14.7	97.5	.582
	Multiparas	19.8				16.5		
Taking bold steps together	Primiparas	18.2	65	.055		14.5	93	.447
	Multiparas	12.0				16.9		
Having fun	Primiparas	16.8	88	.339		16.7	90	.384
	Multiparas	13.8				13.9		
Connecting	Primiparas	13.8	81	.214		14.4	91.5	.422
	Multiparas	17.8				17.0		
Entrusting oneself to the other/ Taking care of the other	Primiparas	12.2	54	.016*	0.43	13.2	72	.092
	Multiparas	19.9				18.5		

* p < .05
r: r of Rosenthal; ES: Effect Size

Table S7. Differences in the mother's dance steps at 6 and 9 months by type of delivery.

Dance steps	Delivery	6 months	Mann–Whitney test		r (ES)	9 months	Mann–Whitney test		r (ES)
		Mean rank	U	p		Mean rank	U	p	
Building together the sense of the ground	Spontaneous	11.8	64	.078		10.7	51.5	.020*	0.42
	C-section	17.6				18.3			
Perceiving one another	Spontaneous	13.0	77	.233		10.1	44.5	.009*	0.47
	C-section	17.0				18.7			
Acknowledging one another	Spontaneous	13.8	85.5	.407		11.4	59.5	.051	
	C-section	17.0				17.9			
Adjusting to one another	Spontaneous	9.6	40	.005*	0.51	7.2	13.5	<.001*	0.71
	C-section	18.9				20.3			
Taking bold steps together	Spontaneous	15.6	103	.948		11.5	60	.047*	0.35
	C-section	15.4				17.8			
Having fun	Spontaneous	13.7	85	.394		9.1	33.5	.002*	0.56
	C-section	16.5				19.2			
Connecting	Spontaneous	12.7	74	.187		11.5	51.5	.021*	0.42
	C-section	17.1				17.8			
Entrusting oneself to the other/ Taking care of the other	Spontaneous	9.0	33	.002*	0.56	13.2	60.5	.048*	0.35
	C-section	19.3				18.5			

* p < .05
r: r of Rosenthal; ES: Effect Size

Table S8. Differences in the infant's dance steps at 6 and 9 months by type of delivery.

Dance steps	Delivery	6 months	Mann–Whitney test		r (ES)	9 months	Mann–Whitney test		r (ES)
		Mean rank	U	p		Mean rank	U	p	
Building together the sense of the ground	Spontaneous	10.8	52.5	.020		14.5	93.5	.616	
	C-section	18.2				16.1			
Perceiving one another	Spontaneous	15.0	99	.812		14.3	91	.558	
	C-section	15.8				16.2			
Acknowledging one another	Spontaneous	9.8	41.5	.006*	0.50	14.3	91.5	.573	
	C-section	18.8				16.2			
Adjusting to one another	Spontaneous	9.8	42	.007*	0.49	9.7	40.5	.006*	0.50
	C-section	18.8				18.9			
Taking bold steps together	Spontaneous	13.1	78.5	.148		12.7	74	.177	
	C-section	16.9				17.1			
Having fun	Spontaneous	14.2	90.5	.527		10.4	48.5	.014*	0.44
	C-section	16.2				18.5			
Connecting	Spontaneous	11.4	59	.026*	0.36	11.5	59.5	.045*	0.35
	C-section	17.9				17.9			
Entrusting oneself to the other/ Taking care of the other	Spontaneous	12.2	68.5	.093		12.6	72.5	.077	
	C-section	17.4				17.2			

* $p < .05$
r: r of Rosenthal; ES: Effect Size

Table S9. Differences in the mother's dance steps at 6 and 9 months by level of education.

Dance steps	Level of Education	6 months	Kruskal–Wallis test		η2 (ES)	9 months	Kruskal–Wallis test		η2 (ES)
		Mean rank	χ2	p		Mean rank	χ2	p	
Building together the sense of the ground	Middle school	3.8	15.2	.002*	0.51	5.8	10.2	.017*	0.34
	High school	18.0				17.7			
	Prof. diploma	24.2				21.5			
	Degree	17.3				17.2			
Perceiving one another	Middle school	10.7	10.5	.015*	0.35	10.3	4.5	.213	
	High school	13.1				17.5			
	Prof. diploma	9.0				10.3			
	Degree	22.1				17.9			
Acknowledging one another	Middle school	9.2	13.1	.004*	0.43	7.2	11.9	.008*	0.40
	High school	12.6				17.1			
	Prof. diploma	10.8				8.0			
	Degree	22.9				20.7			
Adjusting to one another	Middle school	5.8	9.7	.021*	0.32	5.5	10.4	.016*	0.35
	High school	18.4				18.8			
	Prof. diploma	16.0				15.0			
	Degree	18.1				18.1			
Taking bold steps together	Middle school	5.8	14.9	.002*	0.50	9.3	5.2	.155	
	High school	14.5				15.9			
	Prof. diploma	12.8				14.0			
	Degree	22.5				19.0			
Having fun	Middle school	6.3	16.2	.001*	0.54	4.9	12.3	.007*	0.41
	High school	12.9				15.9			
	Prof. diploma	15.0				20.0			
	Degree	23.1				19.6			
Connecting	Middle school	8.3	10.8	.013*	0.36	6.8	14.2	.003*	0.47
	High school	19.1				20.8			
	Prof. diploma	26.0				25.0			
	Degree	13.3				12.9			
Entrusting oneself to the other/ Taking care of the other	Middle school	7.3	12.3	.006*	0.41	7.0	13.9	.003*	0.46
	High school	18.2				16.1			
	Prof. diploma	27.5				29.0			
	Degree	14.3				16.0			

* p < .05

Table S10. Differences in the infant's dance steps at 6 and 9 months by level of education.

Dance steps	Level of Education	6 months Mean rank	Kruskal–Wallis test $\chi2$	p	η2 (ES)	9 months Mean rank	Kruskal–Wallis test $\chi2$	p-value	η2 (ES)
Building together the sense of the ground	Middle school	9.7	7.9	.049*	0.26	17.5	7.2	.066	
	High school	15.4				12.6			
	Prof. diploma	26.5				26.5			
	Degree	15.8				14.1			
Perceiving one another	Middle school	14.5	4.3	.231		14.8	0.2	.970	
	High school	16.6				14.9			
	Prof. diploma	6.2				15.5			
	Degree	17.6				16.5			
Acknowledging one another	Middle school	10.4	4.9	.178		15.3	1.2	.763	
	High school	16.9				13.4			
	Prof. diploma	23.5				15.7			
	Degree	11.9				17.5			
Adjusting to one another	Middle school	9.3	7.4	.060		9.8	3.2	.355	
	High school	16.8				16.3			
	Prof. diploma	25.7				18.0			
	Degree	15.0				17.2			
Taking bold steps together	Middle school	11.5	12.3	.006*	0.41	14.8	5.7	.126	
	High school	12.9				19.8			
	Prof. diploma	26.8				18.5			
	Degree	17.0				11.2			
Having fun	Middle school	7.8	11.8	.008*	0.39	5.5	11.4	.010*	0.38
	High school	15.8				16.6			
	Prof. diploma	28.0				23.0			
	Degree	16.1				18.0			
Connecting	Middle school	9.5	9.3	.025*	0.31	10.5	7.0	.073	
	High school	14.9				16.5			
	Prof. diploma	26.0				26.0			
	Degree	16.5				14.5			
Entrusting oneself to the other/ Taking care of the other	Middle school	8.0	16.4	.001*	0.55	11.5	15.6	.001*	0.52
	High school	11.3				12.7			
	Prof. diploma	24.5				29.0			
	Degree	21.0				16.6			

* $p < .05$

Table S11. Differences in the mother's dance steps at 6 and 9 months by employment status.

Dance steps	Employment status	6 months Mean rank	Kruskal–Wallis test χ2	p	η2 (ES)	9 months Mean rank	Kruskal–Wallis test χ2	p	η2 (ES)
Building together the sense of the ground	Employed	22.7	5.6	.061		16.7	0.5	.762	
	Seeking empl.	12.8				16.0			
	Housewives	15.5				13.6			
Perceiving one another	Employed	18.1	0.8	.654		11.2	1.9	.389	
	Seeking empl.	15.4				16.9			
	Housewives	13.8				16.0			
Acknowledging one another	Employed	20.9	3.0	.222		16.3	7.9	.019*	0.21
	Seeking empl.	14.5				18.8			
	Housewives	13.4				8.3			
Adjusting to one another	Employed	15.8	0.1	.994		11.0	5.4	.067	
	Seeking empl.	15.4				18.9			
	Housewives	15.5				12.0			
Taking bold steps together	Employed	26.3	11.7	.003*	0.35	14.1	0.3	.840	
	Seeking empl.	13.5				16.3			
	Housewives	11.4				14.9			
Having fun	Employed	23.5	6.5	.038*	0.17	15.0	1.9	.385	
	Seeking empl.	14.0				17.3			
	Housewives	12.6				12.2			
Connecting	Employed	16.3	0.9	.645		13.6	0.4	.819	
	Seeking empl.	14.2				16.2			
	Housewives	17.6				15.5			
Entrusting oneself to the other/ Taking care of the other	Employed	13.2	1.1	.580		17.0	1.9	.388	
	Seeking empl.	15.2				13.6			
	Housewives	17.9				18.3			

* $p < .05$

r: r of Rosenthal; ES: Effect Size

Table S12. Differences in the mother's dance steps at 6 and 9 months by postnatal depression.

Dance steps	EPDS	6 months	Mann–Whitney test		r	9 months	Mann–Whitney test		r
		Mean rank	U	p	(ES)	Mean rank	U	p	(ES)
Building together the sense of the ground	Score < 13	16.5	74.5	.360		15.8	88.5	.781	
	Score > 13	13.3				14.8			
Perceiving one another	Score < 13	18.9	24	.001*	0.58	16.6	70.5	.275	
	Score > 13	7.7				12.8			
Acknowledging one another	Score < 13	17.3	57.5	.089		14.5	74	.350	
	Score > 13	11.4				17.8			
Adjusting to one another	Score < 13	16.7	69	.241		16.6	72	.303	
	Score > 13	12.7				13.0			
Taking bold steps together	Score < 13	17.3	56	.079		15.6	91.5	.888	
	Score > 13	11.2				15.2			
Having fun	Score < 13	18.2	38.5	.010*	0.46	18.2	38.5	.010*	0.46
	Score > 13	9.3				9.3			
Connecting	Score < 13	17.7	48	.034*	0.38	15.9	85.5	.681	
	Score > 13	10.3				14.5			
Entrusting oneself to the other/ Taking care of the other	Score < 13	16.6	72	.301		14.8	80.5	.508	
	Score > 13	13.0				17.1			

* p < .05
EPDS. Edinburgh Postnatal Depression Scale
r: r of Rosenthal; ES: Effect Size

Table S13. Differences in the infant's dance steps at 6 and 9 months by postnatal depression.

Dance steps	EPDS	6 months	Mann–Whitney test		r	9 months	Mann–Whitney test		r
		Mean rank	U	p	(ES)	Mean rank	U	p	(ES)
Building together the sense of the ground	Score < 13	16.4	75.5	.373		18.1	39	.008*	0.46
	Score > 13	13.4				9.3			
Perceiving one another	Score < 13	17.1	61	.127		18.3	35	.007*	0.49
	Score > 13	11.8				8.9			
Acknowledging one another	Score < 13	19.1	20	.001*	0.62	18.7	26.5	.002*	0.56
	Score > 13	7.2				7.9			
Adjusting to one another	Score < 13	19.9	1.5	<.001*	0.77	19.3	15.5	<.001*	0.65
	Score > 13	5.2				6.7			
Taking bold steps together	Score < 13	17.3	58.5	.035*	0.30	19.0	21	.001*	0.61
	Score > 13	11.5				7.3			
Having fun	Score < 13	16.8	67.5	.200		18.7	26.5	.002*	0.56
	Score > 13	12.5				7.9			
Connecting	Score < 13	18.1	40.5	.006*	0.45	19.1	18	<.001*	0.63
	Score > 13	9.5				7.0			
Entrusting oneself to the other/ Taking care of the other	Score < 13	17.3	57	.066		17.2	58.5	.036*	0.30
	Score > 13	11.3				11.5			

* p < .05
EPDS. Edinburgh Postnatal Depression Scale
r: r of Rosenthal; ES: Effect Size

Table S14. Correlation between the mother's dance steps at 6 months and perceived social support.

			MSPSS			
			**Family**	**Friends**	**Significant others**	**Total**
**Mother's Dance Steps**	A	Spearman's correlation	.131	-.279	-.169	-.147
		Sign. (two-tailed)	.490	.136	.373	.438
	B	Spearman's correlation	.012	-.264	-.239	-.133
		Sign. (two-tailed)	.950	.158	.203	.483
	C	Spearman's correlation	.222	-.072	-.099	.046
		Sign. (two-tailed)	.239	.705	.603	.809
	D	Spearman's correlation	.175	-.164	-.120	-.083
		Sign. (two-tailed)	.356	.388	.528	.664
	E	Spearman's correlation	.234	-.128	-.110	.037
		Sign. (two-tailed)	.214	.501	.563	.845
	F	Spearman's correlation	.290	-.085	-.009	.091
		Sign. (two-tailed)	.120	.657	.963	.633
	G	Spearman's correlation	-.138	-.495**	-.329	-.376*
		Sign. (two-tailed)	.468	.005	.076	.041
	H	Spearman's correlation	.116	-.224	-.107	-.097
		Sign. (two-tailed)	.543	.235	.575	.611

*. The correlation is significant at the 0.05 level (two-tailed), **. The correlation is significant at the 0.01 level (two-tailed). A. Building together the sense of the ground; B. Perceiving one another; C. Acknowledging one another; D. Adjusting to one another; E. Taking bold steps together; F. Having fun; G. Connecting; H. Entrusting oneself to the other/Taking care of the other. MSPSS. Multidimensional Scale of Perceived Social Support

Table S15. Correlation between the mother's dance steps at 9 months and perceived social support.

			MSPSS			
			**Family**	**Friends**	**Significant others**	**Total**
**Mother's Dance Steps**	A	Spearman's correlation	.295	.012	.145	.093
		Sign. (two-tailed)	.113	.948	.446	.625
	B	Spearman's correlation	.112	-.140	-.131	-.065
		Sign. (two-tailed)	.556	.461	.490	.733
	C	Spearman's correlation	.412*	.216	.046	.231
		Sign. (two-tailed)	.024	.251	.811	.220
	D	Spearman's correlation	.207	-.139	-.114	-.030
		Sign. (two-tailed)	.273	.463	.548	.873
	E	Spearman's correlation	.414*	.122	.225	.258
		Sign. (two-tailed)	.023	.521	.233	.168
	F	Spearman's correlation	.284	-.118	.036	.070
		Sign. (two-tailed)	.128	.534	.850	.712
	G	Spearman's correlation	.109	-.242	-.118	-.110
		Sign. (two-tailed)	.565	.198	.533	.561
	H	Spearman's correlation	.388*	.037	.291	.212
		Sign. (two-tailed)	.034	.844	.118	.260

*. The correlation is significant at the 0.05 level (two-tailed).

A. Building together the sense of the ground; B. Perceiving one another; C. Acknowledging one another; D. Adjusting to one another; E. Taking bold steps together; F. Having fun; G. Connecting; H. Entrusting oneself to the other/Taking care of the other. MSPSS. Multidimensional Scale of Perceived Social Support
